# Effectiveness of Physical Activity Interventions on Cognition, Neuropsychiatric Symptoms, and Quality of Life of Alzheimer’s Disease: An Update of a Systematic Review and Meta-Analysis

**DOI:** 10.3389/fnagi.2022.830824

**Published:** 2022-03-02

**Authors:** Ya-Jing Liang, Qing-Wen Su, Zhi-Ren Sheng, Qiu-Yan Weng, Yan-Fang Niu, Hong-Di Zhou, Chun-Bo Liu

**Affiliations:** ^1^Department of Nursing, The Affiliated Hospital of Medical School, Ningbo University, Ningbo, China; ^2^Department of Health Management Center, The Affiliated Hospital of Medical School, Ningbo University, Ningbo, China; ^3^Department of Neurology, The Affiliated Hospital of Medical School, Ningbo University, Ningbo, China

**Keywords:** physical activity, Alzheimer’s disease, cognition, neuropsychiatric symptoms, quality of life, systematic review, meta-analysis

## Abstract

The topic of physical activity interventions for the treatment of Alzheimer’s disease (AD) has been discussed for decades, but there are still inconsistent views on the effect of its intervention in different studies. With the increase in randomized controlled trials (RCTs), it is necessary to update newly published studies and systematically evaluate the effects of physical activity interventions. Scientific citation databases (e.g., PubMed, EMBASE, etc.) and registration databases (e.g., ISRCTN, CHICTR, etc.) were checked to screen RCTs and systematic reviews of physical activity interventions in AD. Then extract and review the intervention methods and their evaluation results in the included studies. Spearman correlation method was used to test the association between the mean difference (MD) of intervention results and activity time. The Hedges’g method was used to combine continuous data to analyze the standard MD (SMD) of different intervention types or time subgroups. The overall results show that physical activity intervention can improve the cognition, neuropsychiatric symptoms and quality of life (Qol) of AD patients, but the duration of the intervention significantly affected the outcome of the assessment. Subgroup analysis results showed that an intervention duration of 2–5 months had a significant advantage: cognitive function (Minimum Mental State Examination: SMD = 0.47, 95% CI = 0.33 ∼ 0.61, *P* < 0.01), neuropsychiatric symptoms (Neuropsychiatric Inventory: SMD = −0.48, 95% CI = −0.85 ∼−0.11, *P* < 0.01), and quality of life (Qol-AD: SMD = 0.47, 95% CI = 0.23 ∼ 0.71, *P* < 0.01). The systematic review and analysis results of updated RCTs suggested that short-term (2–5 months) physical activity interventions were more beneficial in improving cognitive function, neuropsychiatric symptoms and Qol in patients with AD. And there was no evidence of differences in the effectiveness of different physical activity interventions.

## Introduction

Alzheimer’s disease (AD) is a common type of senile dementia (Lane et al., 2018). Early physical activity intervention has a positive preventive effect on AD high-risk population (De la Rosa et al., 2020), but a review of a prospective cohort study shows that physical activity is associated with reduced risk of vascular dementia, not AD (Norton et al., 2014). At the same time, many randomized controlled studies of physical activities used to improve cognitive impairment, neuropsychiatric symptoms or quality of life in AD (Qol-AD) population have reached inconsistent conclusions. For example, in the results of a recent randomized controlled trial (RCT), 6 months of aerobic exercise significantly slowed down the natural decline of the overall cognitive ability of patients with mild to severe AD, but there was no difference compared with those with general stretching exercise (Yu et al., 2021). However, in another RCT reported by Sobol et al. (2016), aerobic exercise intervention was not found to significantly improve the Minimum Mental State Examination (MMSE) and Neuropsychiatric Inventory (NPI) scores of AD patients, but only improved the patients’ motor function.

Physical activity has a complicated process to improve cognitive ability. An inappropriate physical intervention therapy will reduce the quality of life of AD patients, increase the condition of AD patients and the burden of their caregivers. The benefits of single exercise therapy on the cognitive function and life ability of AD patients was not significantly different from the results of multi-modal interventions (Lamb et al., 2018; de Oliveira et al., 2019). These inconsistent results will interfere with physical therapists in making better rehabilitation programs. Therefore, simply pursuing whether a certain interference factor significantly improves the cognitive or motor function of AD patients is not conducive to the strengthening of favorable factors, but hinders the improvement and formulation of the best rehabilitation program. In addition, the lack of control for confounding factors is also the reason why many studies have reached inconsistent conclusions. Based on a systematic review of previous research results, this study aims to evaluate the effect of physical exercise on improving the cognitive impairment, neuropsychiatric symptoms and Qol of AD patients by adding new RCTs.

## Materials and Methods

The data and information used in this study follow the requirements of the dissemination and application policy of public databases (e.g., PubMed, CNKI, etc.).

### Search Strategy

Keywords include “physical exercise,” “aerobic exercise,” “Alzheimer’s disease,” “cognitive impairment,” “meta-analysis,” “systematic review,” and “neuropsychiatric symptoms.” Scientific citation databases include PubMed, EMBASE, CNKI, Web of Science and Cochrane Library. Registered databases include ISRCTN, ClinicalTrials, CHICTR and ANZCTR. The database retrieval time occurred on 1 October 2021. Searching formula include (i) ((systematic review[Title/Abstract]) OR (meta-analysis[Title/Abstract])) AND (Alzheimer’s disease[Title/Abstract]) AND ((aerobic exercise[Title/Abstract]) OR (physical exercise[Title/Abstract])), (ii) ((aerobic exercise[Title/Abstract]) OR (physical exercise [Title/Abstract])) AND (dementia[Title/Abstract]) AND ((cognitive impairment[Title/Abstract]) OR (neuropsychiatric symptoms[Title/Abstract]) OR (quality of life[Title/Abstract]) OR (physical function[Title/Abstract])).

### Inclusion and Exclusion Criteria

Inclusion criteria: (1) The type of experimental design is a RCT; (2) The literature clearly states that the subjects participating in the experiment are AD patients; (3) At least two assessment results of cognitive impairment, neuropsychiatric symptoms and quality of life are included; (4) The cognitive impairment assessment scale is mainly based on Mini Mental State Examination (MMSE) or Alzheimer’s Disease Assessment Scale - Cognition (ADAS-cog); (5) Physical activities include aerobic exercise, treadmill, cycling, fast walking, balance and strength training; and (6) Have a detailed exercise plan and time control, and exercise regularly for at least 2 months.

Exclusion criteria: (1) Participants were patients with vascular dementia or other types of dementia; (2) The intervention results did not report the evaluation data of the scale, or did not record the difference of the results; (3) If there are cases of loss to follow-up or withdrawal from training, the baseline evaluation results of the subjects who have completed the training period are required; and (4) Observational retrospective research.

### Data Extraction

According to the PICOS principle, the population, intervention, comparison, outcome and study design information were extracted. In addition, the newly included literature was evaluated with reference to the Cochrane risk bias tool. The evaluation items included random cohort allocation, allocation hiding, double blinding of participants and staff, blinded evaluation results, complete report data, selective reporting and others. Each item is divided into three levels: unclear, low-risk, and high-risk. The higher the level, the more limited the evaluation of evidence in the corresponding research. Data extraction (Liang, YJ and Su, QW) and bias risk assessment (Sheng, ZR and Weng, QY) were performed by two different authors, and the results were reviewed by a third author (Niu, YF).

### Statistical Analysis

First, this study conducted a retrospective analysis of the studies included in the previously published systematic review or meta-analysis. Spearman method was used to analyze the correlation between assessment results and different activity time, including baseline and the intervention time or follow-up time. Then, a meta-analysis was performed with the newly added RCTs to test whether the results were consistent with those before the update. In this study, the Hedges’g method was used to combine standardized mean difference (SMD) between groups to reduce the deviation caused by the small sample size in each RCT. All relevant analysis processes were completed in R-4.1.0 (R Foundation for Statistical Computing, Vienna, Austria).

## Results

### Basic Characteristics of Updated Literature

**FIGURE 1:**
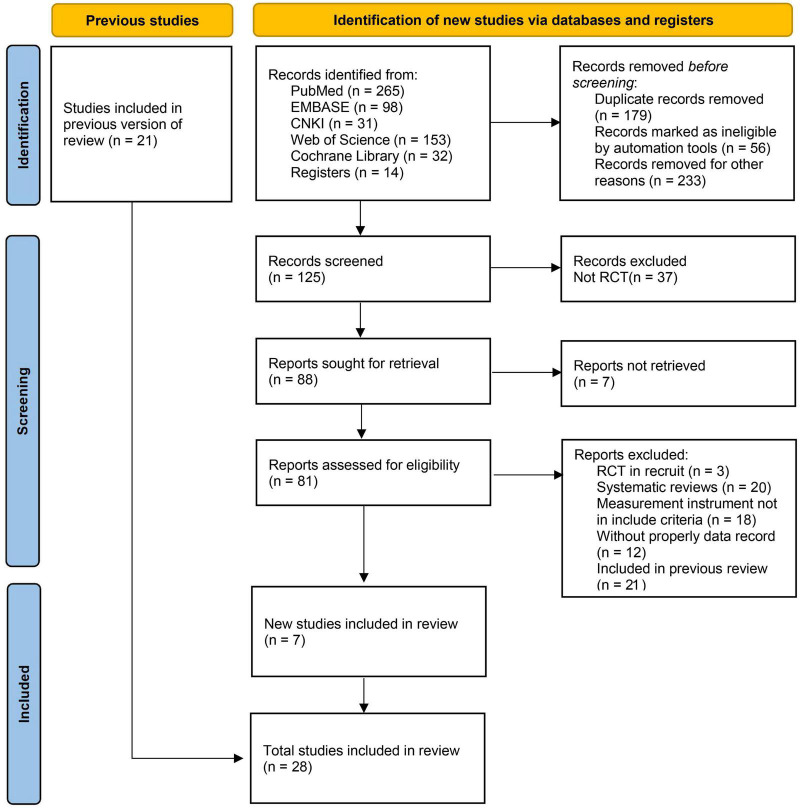
Flow diagram of literature retrieval and screening (PRISMA 2020 statement).

According to the literature retrieval and screening process of PRISMA (2020) (Page et al., 2021; Figure 1), in addition to 21 studies in previously reported systematic reviews (Cammisuli et al., 2018; Du et al., 2018; Liang et al., 2018; Jia et al., 2019), this study updated 7 related articles (Table 1), two of which were retrieved from CNKI database of China. All included literature were provided as Supplementary Data Sheet 1.

**TABLE 1:** Basic information of updated RCTs.

Author (Year) Country	Age (year), mean (SD)*	Sample*	Content of interventions		Exercise time and cycle	Diagnostic criteria	Measurement instrument	Registered number	Outcomes	Bias risk@
			EG	CG						
Yu et al. (2021) United States	EG:77.4 (6.6) CG:77.5 (7.1)	EG:64 CG:32	Cycling at 50–75% of heart rate reserve (HRR)	Stretching and range-of-motion, <20% of HRR	20–50 min a session, 3 times a week, for 6 months	CDR, MMSE	ADAS-cog,composite scores	NCT019 54550	The 6 month change in ADAS-cog sinificnatly less than the natural increase in AD	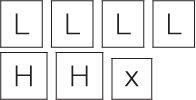
de Oliveira et al. (2019) Brazil	EG:81.2 (8.9) CG:77.5 (8.1)	EG:12 CG:7	Multimodal training: balance, aerobic, and strength training and stretching	Clinical follow-up, without any physical training	60 min a session, twice a week, for 4 month	DSM-IV, CDR, MMSE	MMSE,CDR,clock drawing test (CDT),8-foot up and go test	NA	Physical exercise program did not improve cognition, mobility and executive function in AD patients.	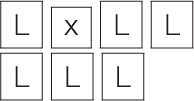
Lamb et al. (2018) United Kingdom	EG:76.9 (7.9) CG:78.4 (7.6)	EG:329 CG:165	Moderate to hard intensity cycling, hold dumb bells	Health and social care	60–90 min a session, twice a week, for 4 month	DSM-IV, MMSE	MMSE,ADAS-cog,EQ-5D-Qol,NPI	ISRCTN10 416500	The exercise training program improved physical fitness without slowing cognitive impairment in Alzheimer’s patients.	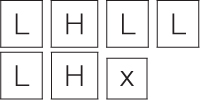
Sobol et al. (2016) Denmark	EG:69.8 (7.4) CG:71.3 (7.3)	EG:107 CG:93	Moderate-to-high–intensity aerobic exercise on ergometer bicycle, cross trainer, and treadmill	Usual treatment	60 min a session, 3 times a week, for 4 months	NINDS-ADRDA	SDMT,NPI,TUG, 10-m walk test	NCT016 81602	Aerobic exercise showed significant positive effects on physical performance.	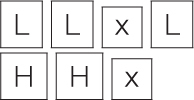
Aguiar et al. (2014) Brazil	EG:78.6 (8.4) CG:74.7 (7.4)	EG:17 CG:17	Walking, stair-climbing,resistance and dynamic balance training, and Rivastigmine Transdermal Patch (RTP)	RTP alone	40 min a session, twice a week, for 6 months	NA	MMSE,Qol-AD,TUG	NCT011 83806	There was a significant improvement in QOL of physical exercise group. There was no difference in cognitive function between the two groups.	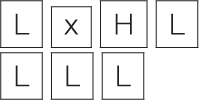
Qing et al. (2020) China	EG:74 (11) CG:70 (11)	EG:30 CG:30	Aerobic exercise,60–80% of HRR	Health education	40 min a session, 3 times a week, for 3 months	NINDS-ADRDA	MMSE,NPI,Qol-AD,ASCS-ADL,BBS	NA	MMSE, ADCS-ADL scores are significantly higher in aerobic exercise group at 3 months follow-up	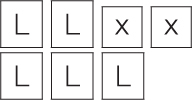
Chunhong et al. (2015) China	EG:70.7 (7.4) CG:70.2 (8.5)	EG:27 CG:30	Aerobic exercise,50–70% of HRR	Usual treatment	60–90 min, 3 times a week, for 4 month	DSM-IV, MMSE	MMSEQol-ADASCS-ADL	NA	Aerobic training therapy can significantly improve the cognition, ADL and quality of life.	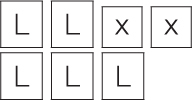

*EG, experiment group; CG, contral group; SD, standerd deviation; CDR, Clinical Dementia Rating; SDMT, Symbol Digit Modalities Test; NPI, Neuropsychiatric Inventory; MMSE, Minimum Mental State Examination; ADAS-cog, Alzheimer’s Disease Assessment Scale - Cognition; Qol-AD, Quality of Life - Alzheimer’s Disease; ADCS-ADL, Alzheimer’s Disease Cooperative Study - Activity of Daily Living; BBS, Berg Balance Scale; TUG, Time Up and Go test; DSM-IV, Diagnostic and Statistical Manual of Mental Disorders, fourth edition; NINDS-ADRDA, National Institute of Neurological and Communicative Disorders and Stroke and the Alzheimer’s Disease and Related Disorders Association; NA, Not available. @bias risk evaluation. Symbol means: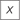 unclear, 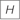 high risk, and 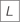 low risk. The seven evaluation items included random cohort allocation, allocation hiding, double blinding of participants and staff, blinded evaluation results, complete report data, selective reporting and others. * record at baseline.*

### Systematic Review of Previous Reports

**FIGURE 2:**
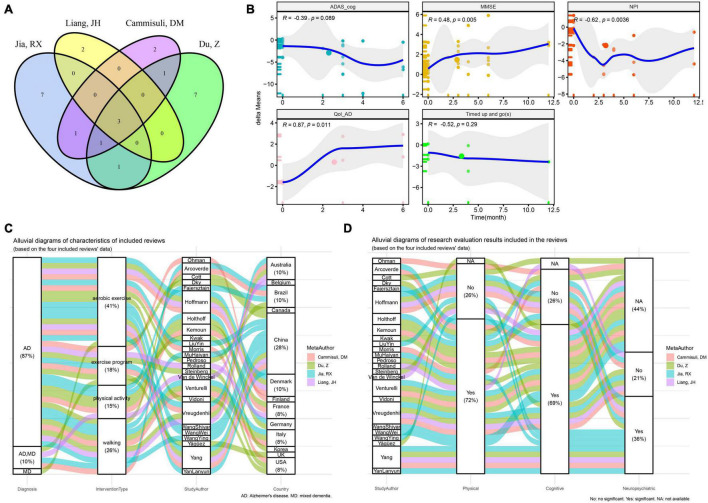
Overview of studies included in the previous reported meta-analysis of physical activity interventions for cognitive function, neuropsychiatric symptoms and quality of life in Alzheimer’s disease. **(A)** Venn plot of the included studies in the four previously reported systematic reviews. **(B)** Spearman correlation test between the intervention time and the delta-means of ADAS_cog, MMSE, NPI, and Qol-AD. **(C)** Distribution of dementia and corresponding intervention types and counties of all included studies. **(D)** Distribution of intervention types and corresponding conclusions of all included studies (ADAS_cog, Alzheimer’s Disease Assessment Scale - Cognition; MMSE, Minimum Mental State Examination; NPI, Neuropsychiatric Inventory Questionnaire; Qol-AD, quality of life - Alzheimer’s Disease; delta-means, the difference between the results of each reevaluation and the baseline in each study).

After reviewing 25 different documents included in 4 systematic reviews (Cammisuli et al., 2018; Du et al., 2018; Liang et al., 2018; Jia et al., 2019), it was found that 4 of them (Van de Winckel et al., 2004; Kwak et al., 2008; Miu et al., 2008; Arcoverde et al., 2014) recruited patients with other types of dementia, so they were not included in this study. As shown in Figure 2A, although these four systematic reviews were published in 2018 or 2019, there are still quantitative differences and omissions in the included literature.

Subsequently, in the 21 RCT results, the differences in the MMSE, ADAS-cog, NPI and Qol-AD of AD patients with different physical activity interventions relative to the baseline scores are calculated as dependent variable “delta means.” The results of Spearman correlation analysis of fitting the time regression curve (Figure 2B) show that MMSE (*R* = 0.48, *P* < 0.01), NPI (*R* = −0.62, *P* < 0.01) and Qol-AD (*R* = 0.87, *P* < 0.05) have a significant correlation with the duration of physical activity.

Reviewing the characteristics of interventions included in the study (Figure 2C), 41% of the included studies assessed the intervention effects of aerobic exercise, while 26, 18, and 15% of the included studies assessed the intervention effects of walking, exercise programs, and physical activity, respectively. Among them, 28% of the research results are from AD patients in China, and six studies of the research results reported in Chinese are from the CNKI database. In this study, the results from the CNKI database were used as a subgroup retrospective analysis. It is worth noting that Spearman correlation analysis showed that there was a significant correlation between exercise intervention and the duration of activity. However, no significant correlation results were found in subgroup of other databases (Table 2).

**TABLE 2:** Correlation test of means change difference (delta-means) at different time points.

Subgroup	Cat.	ADAS-cog	MMSE	NPI	Qol-AD	TUG
All	*n*	20	33	20	7	6
	T	0,2,3,4,6	0,2,3,4,6,12	0,2,3,4,6,12	0,3,6	0,3,12
	*Rho*	−0.39	0.48	−0.62	0.87	−0.52
	*P*	0.089	**0.005**	**0.0036**	**0.011**	0.29
CNKI database	*n*	14	16	8	3	.
	*T*	0,2,3,6	0,2,3,4,6	0,2,3,4,6	0,3,6	.
	*Rho*	−0.54	0.75	−0.76	1	.
	*P*	**0.045**	** <0.001**	**0.028**	0.33	.
Other databases	*n*	6	17	12	4	6
	*T*	0,3,4	0,3,4,6,12	0,3,4,6,12	0,3,6	0,3,12
	*Rho*	−0.4	0.32	−0.5	0.95	−0.52
	*P*	0.43	0.21	0.1	0.051	0.29
Aerobic exercise	*n*	18	20	12	5	2
	*T*	0,2,3,4,6	0,2,3,4,6	0,2,3,4,6	0,3,6	0,4
	*Rho*	−0.39	0.58	−0.74	0.95	−1
	*P*	0.11	**0.0069**	**0.0056**	**0.014**	1
Other exercise	*n*	2	13	8	2	4
	*T*	0,4	0,3,4,6,12	0,3,6,12	0,6	0,3,12
	*Rho*	−1	0.36	−0.25	1	−0.74
	*P*	1	0.23	0.56	1	0.26

*MMSE, Minimum Mental State Examination; NPI, Neuropsychiatric Inventory; Qol-AD, Quality of Life - Alzheimer’s Disease; ADAS-cog, Alzheimer’s Disease Assessment Scale - Cognition; n, total number of assessment points; T, assessemnt time point (months); Rho, Spearman correlation coefficient; P, P value; TUG, timed up and go (s); CNKI, China national knowledge infrastructure. Bold value indicate a P-value less than 0.05.*

We found that the aforementioned six studies all used aerobic exercise as an intervention method (Figure 2C). Stratified analysis of aerobic exercise and other types of exercise as subgroups showed significant correlation results only in the aerobic exercise intervention group, while there was no significant difference in other exercise intervention conditions (Table 2). In addition, as shown in Figure 2D, 72% of the studies concluded that physical activity intervention is effective on the functional ability of AD patients, of which about 59% (69% of the total) also positively improved cognition. Only 36% of studies indicated improvement in neuropsychiatric symptoms.

### Updated Results of Meta-Analysis

**FIGURE 3:**
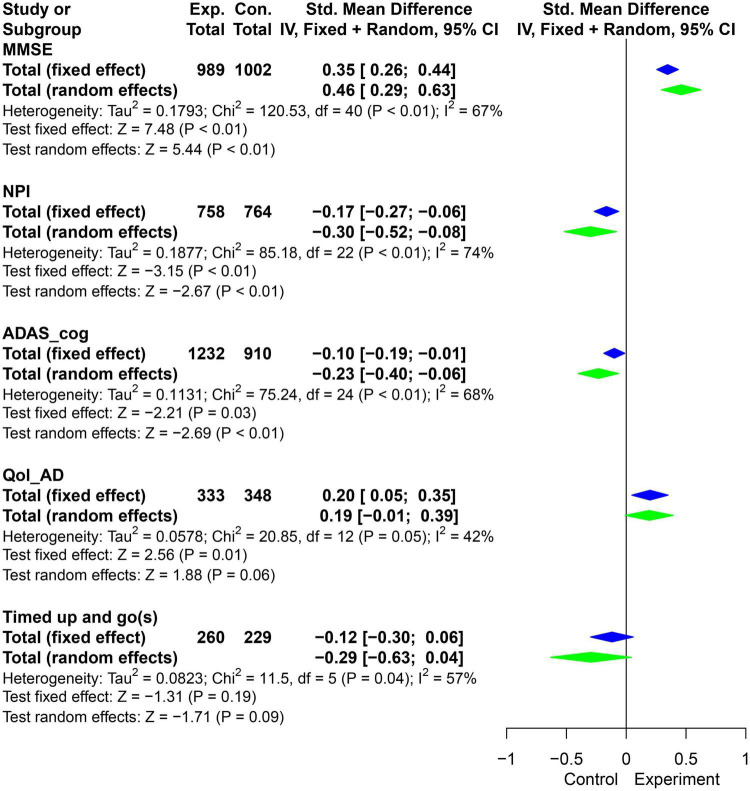
Pooled SMD forest plot of the results of physical activity intervention on MMSE, ADAS_cog, NPI, and Qol-AD. (SMD, standard mean difference; ADAS_cog, Alzheimer’s Disease Assessment Scale - Cognition; MMSE, Minimum Mental State Examination; NPI, Neuropsychiatric Inventory Questionnaire; Qol-AD, Quality of Life - Alzheimer’s Disease).

By adding the new RCT results (Figure 3), ignoring the influence of confounding factors such as training time, types of physical activities, assistance from professional therapists and types of caregivers, the development of planned and purposeful physical activities can improve the cognitive impairment (MMSE: SMD = 0.46, 95% CI = 0.29 ∼ 0.63, *P* < 0.01; ADAS-cog: SMD = −0.23, 95% CI = −0.4 ∼−0.06, *P* < 0.01), neuropsychiatric symptoms (NPI: SMD = −0.3, 95% CI = −0.52 ∼−0.08, *P* < 0.01) and quality of life (Qol-AD: SMD = 0.2, 95% CI = 0.05 ∼ 0.35, *P* < 0.05) of AD patients.

**FIGURE 4:**
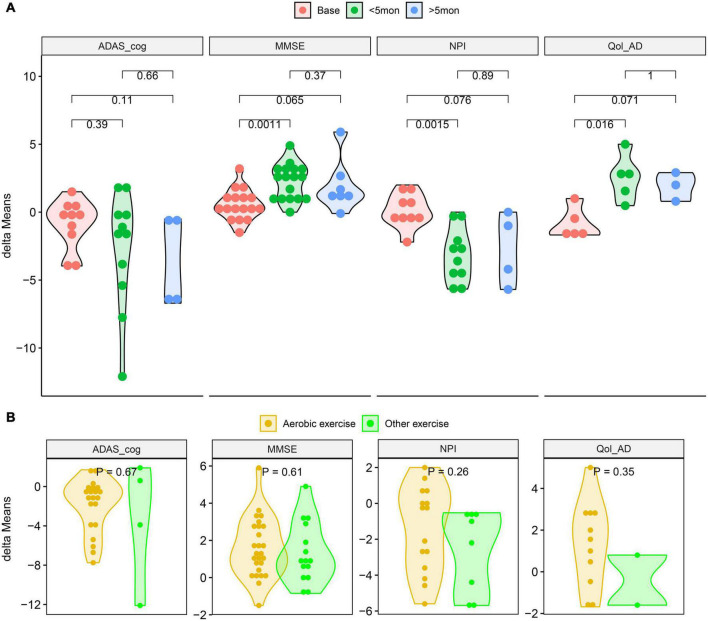
Compared the delta-means generated between baseline and intervention outcomes. **(A)** *T*-tests of the delta-means of MMSE, ADAS_cog, NPI and Qol-AD at different intervention times. **(B)** *T*-tests of the delta-means between aerobic and other exercise of MMSE, ADAS_cog, NPI, and Qol-AD. (ADAS_cog, Alzheimer’s Disease Assessment Scale - Cognition; MMSE, Minimum Mental State Examination; NPI, Neuropsychiatric Inventory Questionnaire; Qol-AD, Quality of Life - Alzheimer’s Disease; delta-means, the difference between the results of each reevaluation and the baseline in each study).

The results showed that there was significant heterogeneity in the combined effects of MMSE, NPI and ADAS-cog (I^2^ > 50%, *P* < 0.05). Meta regression (Table 3) suggested that activity time was one of the reasons for the heterogeneity of MMSE and Qol-AD (*P* < 0.05). In addition, grouped by average physical activity time for 5 months, the *t*-test results showed that the difference of delta-means in the observation indicators (MMSE, NPI, and Qol-AD) had obvious advantages in the intervention group with less than 5 months (Figure 4A). Therefore, it is necessary to further explore the effects of different physical activities and intervention time on AD patients.

**TABLE 3:** Updated subgroup stratified meta-analysis results.

Groups	Subgroups	Cat.	*k*	SMD	[LCI;UCI]	*Z*	*P*	I^2^ (%)	Meta-reg
MMSE	> 5mon	Fixed	24	0.249	[0.128;0.369]	4.05	** <0.01**	73.60	∼Time: β0 = 0.274 β1 = 0.067 *z* = 2.484 *P* = 0.013
		Random		0.392	[0.144;0.640]	3.1	** <0.01**		
	Aerobic	Fixed	14	0.205	[0.068;0.342]	2.95	** <0.01**	75.70	
		Random		0.331	[0.041;0.621]	2.24	**0.03**		
	Other	Fixed	10	0.399	[0.144;0.654]	3.07	** <0.01**	71.80	
		Random		0.505	[0.014;0.997]	2.02	**0.04**		
	<5mon	Fixed	17	0.471	[0.334;0.608]	6.73	** <0.01**	42.20	
		Random		0.553	[0.361;0.745]	5.65	** <0.01**		
	Aerobic	Fixed	12	0.427	[0.279;0.573]	5.69	** <0.01**	48.60	
		Random		0.51	[0.292;0.723]	4.63	** <0.01**		
	Other	Fixed	5	0.767	[0.386;1.147]	3.95	** <0.01**	0.00	
		Random		0.767	[0.386;1.147]	3.95	**<0.01**		
ADAS-cog	> 5mon	Fixed	14	−0.094	[−0.213;0.025]	−1.54	0.12	74.00	∼Time: β0 = −0.227 β1 = −0.003 *z* = −0.139 *P* = 0.889
		Random		−0.235	[−0.491;0.019]	−1.81	0.07		
	Aerobic	Fixed	12	−0.147	[−0.291; −0.003]	−2.01	**0.04**	76.70	
		Random		−0.287	[−0.601;0.027]	−1.79	0.07		
	Other	Fixed	2	0.024	[−0.190;0.240]	0.23	0.82	4.90	
		Random		0.018	[−0.216;0.253]	0.15	0.88		
	<5mon	Fixed	11	−0.106	[−0.237;0.025]	−1.58	0.11	60.40	
		Random		−0.24	[−0.473; −0.008]	−2.03	**0.04**		
	Aerobic	Fixed	9	−0.198	[−0.364; −0.033]	−2.36	**0.02**	43.20	
		Random		−0.251	[−0.485; −0.017]	−2.1	**0.04**		
	Other	Fixed	2	0.052	[−0.163;0.269]	0.48	0.63	87.30	
		Random		−0.281	[−1.240;0.678]	−0.57	0.57		
NPI	> 5mon	Fixed	13	−0.067	[−0.201;0.067]	−0.98	0.33	71.20	∼Time: β0 = −0.159 β1 = −0.048 *z* = −1.411 *P* = 0.158
		Random		−0.173	[−0.446;0.100]	−1.24	0.21		
	Aerobic	Fixed	8	0.039	[−0.127;0.206]	0.46	0.64	0.00	
		Random		0.039	[−0.127;0.206]	0.48	0.64		
	Other	Fixed	5	−0.265	[−0.492; −0.038]	−2.29	**0.02**	88.30	
		Random		−0.824	[−1.617; −0.030]	−2.04	**0.04**		
	<5mon	Fixed	10	−0.303	[−0.463; −0.143]	−3.72	** <0.01**	76.70	
		Random		−0.478	[−0.845; −0.111]	−2.56	** <0.01**		
	Aerobic	Fixed	7	−0.277	[−0.456; −0.097]	−3.02	** <0.01**	0.00	
		Random		−0.277	[−0.456; −0.097]	−3.02	** <0.01**		
	Other	Fixed	3	−0.402	[−0.752; −0.053]	−2.26	**0.02**	94.40	
		Random		−1.795	[−4.328;0.738]	−1.39	0.16		
Qol-AD	> 5mon	Fixed	8	0.015	[−0.181;0.212]	0.15	0.88	24.20	∼Time: β0 = −0.058 β1 = 0.102 *z* = 2.923 *P* = 0.004
		Random		0.013	[−0.214;0.239]	0.11	0.91		
	Aerobic	Fixed	6	0.03	[−0.185;0.245]	0.27	0.79	36.60	
		Random		0.027	[−0.243;0.299]	0.2	0.84		
	Other	Fixed	2	−0.057	[−0.535;0.420]	−0.24	0.81	19.10	
		Random		−0.057	[−0.588;0.473]	−0.21	0.83		
	<5mon	Fixed	5	0.471	[0.231;0.710]	3.85	** <0.01**	0.00	
		Random		0.471	[0.231;0.710]	3.85	** <0.01**		
	Aerobic	Fixed	5	0.471	[0.231; 0.710]	3.85	** <0.01**	0.00	
		Random	5	0.471	[0.231; 0.710]	3.85	** <0.01**	0.00	

*MMSE, Minimum Mental State Examination; NPI, Neuropsychiatric Inventory; Qol-AD, Quality of Life - Alzheimer’s Disease; ADAS-cog, Alzheimer’s Disease Assessment Scale - Cognition; Cat., category; SMD, standard mean difference with Hedges’ g as effect measure; LCI, lower confidence interval; UCI, upper confidence interval; Z,P, statistic and p-value in Hedges’g test; I^2^, heterogeneity statistic. Bold value indicate a P-value less than 0.05.*

The results of the subgroup analysis are shown in Table 3. All of the exercise intervention groups “< 5mon” have significant significance, and the differences in the aerobic training subgroups are particularly worthy of attention. However, in all intervention time periods, the advantages in delta-means of aerobic exercise was not significantly different from other exercises (Figure 4B).

## Discussion

Alzheimer’s disease is a common type of dementia in the elderly, but it is not an inevitable result of aging. It is estimated that the total number of dementia patients will reach 100 million by 2050 (Breijyeh and Karaman, 2020). In addition to genetic, environmental and head trauma factors, obesity, hypertension and hyperlipidemia caused by poor lifestyles are also risk factors for AD (Risk, 2019). The prevention and treatment of AD has gone through decades of exploration and attempts. Early clinical trials have also reported many effective pharmacological and non-pharmacological treatments (Breijyeh and Karaman, 2020). Common non-pharmacological interventions such as music therapy (Gómez and Gómez, 2017; Lyu et al., 2018), multisensory stimulation (Han et al., 2017; Maseda et al., 2018) and exercise therapy (Hoffmann et al., 2015; Vidoni et al., 2019) have yielded considerable improvements in cognitive impairment or ability to live daily in a number of small-scale RCTs.

For senile AD caused by multiple factors, the impact of various physical activity interventions on the patient’s cognition and daily activities has a complex process of change, such as thinking judgment, balance control, and emotional management (Brasure et al., 2018). The long-term care of AD patients not only brings psychological and physical burdens to informal caregivers (Cheng, 2017; Frias et al., 2020), but also affects the benefits of treatment. Including the treatment process of the disease and neuropsychiatric symptoms, the negative impact on the caregiver will reduce the self-cognition and evaluation of Qol in AD patients (Cheng, 2017). This may explain that the MMSE, NPI, and Qol-AD assessment results of AD patients tend to deteriorate again after a long period of physical exercise intervention. For example, in our subgroup analysis results, the physical activity intervention over 5 months in present study did not improve the neuropsychiatric symptoms and Qol of AD patients compared with the “< 5mon” intervention group.

Moreover, AD is a progressive degeneration of nerve cells caused by various factors such as age, and there is still no officially recognized pharmacological or non-pharmacological pathway for the plasticity of nerve cell function (Cummings et al., 2019). Currently, only two classes of drugs including cholinesterase inhibitors (Saxena and Dubey, 2019) and N-methyl D-aspartate (NMDA) antagonists (Chang et al., 2020) have been approved by the Food and Drug Administration (FDA) for the treatment of AD. But these two drugs can only improve the symptoms of AD and are not used to cure or prevent AD. The cause of this result may be irreversible damage or aging of nerve cells, cholinergic system defects or neuronal calcium homeostasis disorders (Bordji et al., 2011). And there is still no consensus on the mechanism of AD syndrome.

Due to the limitations of pharmacological therapy, the treatment and prevention of AD will become a protracted battle, which will not only bring economic burden to the family, but also pose a huge challenge to the social health and healthcare system. Especially in developing countries with large populations, such as China, it is entering the aging stage, which brings huge challenges to the social health management system (Fang et al., 2020). No matter the current active pharmacological research targeting neurotransmitter or receptor system (Bordji et al., 2011; Cummings et al., 2019; Sharma et al., 2020) or the multi-mode intervention with physical activity and cognitive stimulation (Silva et al., 2019; De la Rosa et al., 2020), these treatments or interventions still have no effect on the plasticity of neuronal function in AD patients. Therefore, as a common disease in the elderly, the prevention and treatment of AD will still be a long way to go.

In short, as AD patients and close caregivers, it is very important to contact and learn some recreational activities that make their body and mood feel relaxed. In a systematic review of longitudinal observational studies, it was pointed out that leisure-time activity had a protective effect on patients with AD, while work-related physical activity did not (Stephen et al., 2017). Moreover, relaxed and pleasant voluntary physical activity is beneficial to the improvement of brain cognitive function and psychological health (Ma, 2008), promoting close relationship with caregivers, and can also avoid the destructive behavior of patients with severe AD (Cheng, 2017). This kind of relaxed and voluntary physical activity is valuable for reducing the risk of AD and maintaining the long-term effects of exercise intervention.

Some limitations of this study need to be pointed out, (i) this systematic review did not include studies of types other than RCTs, (ii) some RCT studies for which data were not available were not included in the qualitative analysis, and (iii) few studies indicated that magnetic resonance imaging (MRI) and serological testing (e.g., vitamin-B12) were used in the auxiliary diagnosis of AD patients. Furthermore, increasing the number of RCTs and the sample size is important to enhance the reliability of the results.

## Conclusion

In the updated meta-analysis, planned and professional physical activity has a positive effect on improving the cognitive ability, neuropsychiatric symptoms and Qol of AD patients. This considerable benefit is especially significant in short-term (2–5 months) intervention activities. In short, in addition to all appropriate health-benefit lifestyle or diet recommendations, the current evidence is insufficient to provide specific interventions on the type, frequency, intensity, or duration of physical activity that may prevent and treat AD.

## Data Availability Statement

The original contributions presented in the study are included in the article/Supplementary Material, further inquiries can be directed to the corresponding authors.

## Author Contributions

Y-JL, Q-WS, Z-RS, and Y-FN contributed to the study design, analysis, and interpretation of data. Q-YW and H-DZ searched the database and extracted data. Z-RS and Q-YW performed statistical analysis. Y-JL, Q-WS, and C-BL drafted and prepared the manuscript. All authors approved the final manuscript.

## Conflict of Interest

The authors declare that the research was conducted in the absence of any commercial or financial relationships that could be construed as a potential conflict of interest.

## Publisher’s Note

All claims expressed in this article are solely those of the authors and do not necessarily represent those of their affiliated organizations, or those of the publisher, the editors and the reviewers. Any product that may be evaluated in this article, or claim that may be made by its manufacturer, is not guaranteed or endorsed by the publisher.
