# Supplementary material for: Effectiveness of Physical Activity Interventions on Cognition, Neuropsychiatric Symptoms, and Quality of Life of Alzheimer’s Disease: An Update of a Systematic Review and Meta-Analysis
Source: Front Aging Neurosci. 2022 Mar 2;14:830824. doi: 10.3389/fnagi.2022.830824 (PMC8926300; doi:10.3389/fnagi.2022.830824)
Supplement: Supplementary file 1 [file Data_Sheet_1.docx]

Supplementary references:

1. Included from four previously published systematic review and meta-analysis(2, 4-7, 9-12, 14-19, 23-28):
2. Updated randomized controlled experiments (RCTs)(1, 3, 8, 13, 20-22):
3. RCTs retrieved from CNKI database(21-28):

| **Basic information of included RCTs** | | | | | | | | | | |
| --- | --- | --- | --- | --- | --- | --- | --- | --- | --- | --- |
| Author (Year)  Country | Age (year), mean(SD)* | Sample  size* | Content of interventions | | Exercise time and cycle | Diagnostic criteria | Measurement instrument | Registered number | Outcomes | Bias risk@ |
|  |  |  | EG | CG |  |  |  |  |  |  |
| Aguiar [1] (2014)  Brazil | EG:78.6(8.4) CG:74.7(7.4) | EG:17 CG:17 | walking, stair-climbing, resistance and dynamic balance training, and Rivastigmine Transdermal Patch (RTP) | RTP alone | 40mins a session, twice a week, for 6 months | NA | MMSE, Qol-AD, TUG | NCT01183806 | There was a significant improvement in QOL of physical exercise group. There was no difference in cognitive function between the two groups. |  |
| Cott[2]  (2002)  Canada | EG:83.2(8.2)  CG:79.8(8.3) | EG:30 CG:19 | The walk-and-talk intervention was done in the corridors of the residents’ units and consisted of supervised walking in pairs. | control group who did not receive either intervention | 30-min  walk-and-talk session 5 times a week, over 16 weeks. | MMSE | MMSE,  FACS,  LPRS | NA | Residents who received the walk-and-talk intervention did not demonstrate statistically significant differences in the outcome variables measured post-test when compared with residents who received no intervention, even after controlling for individual differences. |  |
| de Oliveira [3] (2019)  Brazil | EG:81.2(8.9) CG:77.5(8.1) | EG:12 CG:7 | multimodal training: balance, aerobic, and strength training and stretching | clinical follow-up, without any physical training | 60mins a session,  twice a week, for 4 month | DSM-IV, CDR,  MMSE | MMSE, CDR, clock drawing test (CDT), 8-foot up and go test | NA | Physical exercise program did not improve cognition, mobility and executive function in AD patients. |  |
| Fajersztajn[4]  (2008)  Brazil | EG:78.4 CG:76.4 | EG:5 CG:5 | physical activities with stimulation of balance and mobility through functional exercises. (e.g. muscle stretching and joint movement, postural transference of objects from  one place to another) | Non-Intervention | Intervention took place over a 12-week period, with a single 1-hour session per week. | DSM-IV, CDR,  MMSE | MMSE,  NPI, ADL,  TUG,  BBS | NA | The functional task physical activity program was efficient in functional balance improvement and also appeared to prevent mobility decline. |  |
| Hoffmann[5]  (2015)  Denmark | EG:69.8(7.4) CG:71.3(7.3) | EG:107 CG:93 | 3 × 10 min on an ergometer bicycle, cross trainer, and treadmill with 2–5 min rest between. The target intensity was 70–80% of maximal HR (MHR). | usual treatment | 60 min of supervised moderate-to-high intensity aerobic exercise three times weekly for 16 weeks. | NINDS-  ADRDA,  MMSE | MMSE,  NPI,  EQ-5D,  SDMT,  ADAS-Cog | Danish Data Protection Agency  (ID No.  30-0718) | no significant differences between intervention and control groups in change from baseline of SDMT, other cognitive tests, quality of life, or activities of daily living. |  |
| Holthoff[6]  (2015)  Germany | EG:72.4(4.3) CG:70.7(5.4) | EG:15 CG:15 | motor-assisted or active resistive training of the legs as well as changes in direction (forward, reverse) every 5 minutes. | health and social care | three times a week for 30 minutes, testing  by a blinded psychologist at baseline, 12 and 24 weeks. | NINDS-  ADRDA,  CDR,  MMSE | MMSE,  NPI, ADL | NCT02196545 | Patients in the intervention group improved during the intervention and returned to initial performance at week 12 whereas the controls revealed continuous worsening. |  |
| Kemoun[7]  (2010)  France | 81.8(5.3) | EG:20 CG:18 | articular mobilization and muscle stimulation. walking, equilibrium and stamina (e.g. walking by striding over boards, going up a step, zigzagging). | did not practice any physical activities | three 1-hour sessions per week covered a period of 19 weeks. | DSM-IV, MMSE | MMSE,  ERFC | NA | physical activity programme can slow cognitive decline and improve quality of walking |  |
| Lamb [8] (2018)  UK | EG:76.9(7.9) CG:78.4(7.6) | EG:329 CG:165 | moderate to hard intensity cycling, hold dumb bells | health and social care | 60-90mins a session, twice a week, for 4 month | DSM-IV, MMSE | MMSE, ADAS-cog, EQ-5D-Qol, NPI | ISRCTN10416500 | The exercise training program improved physical fitness without slowing cognitive impairment in Alzheimer's patients. |  |
| Morris[9]  (2017)  USA | EG:74.4(6.7) CG:71.4(8.4) | EG:39 CG:37 | Aerobic exercise targeting duration of 150min per week, HR zones were gradually increased from 40–55% to 60–75% | core strengthening, resistance bands, modified tai chi or yoga | 150 minutes per week of aerobic exercise for 26-weeks | MCI,  CDR,  MMSE | DAD,  CSD,  peak VO2, brain volumes | NCT01128361 | Aerobic exercise in early AD is associated with benefits in functional ability. |  |
| Öhman[10]  (2016)  Finland | EG:77.7(5.4)  EG:78.3(5.1) CG:78.1(5.3) | EG:70  EG:70 CG:70 | Rowing machine, pedal exerciser, Nordic walking outdoors, dancing.  Walking on balance  beam or line, trampoline jumping, picking up items from floor, training with bouncing ball, climbing a ladder,  rising from floor | routine medical care | 1 hour twice a week for 12 months | NINDS-  ADRDA,  CDR,  MMSE | VF,  CDT,  MMSE | NA | no significant differences between the groups were detected at 12-month follow-up when analyses were adjusted for age, sex, and CDR. |  |
| Pedroso[11]  (2012)  Brazil | EG:78.3(7.4) CG:77.5(6.9) | EG:10 CG:11 | motor task (bouncing a ball, walking, doing exercises with weights). sensory stimuli (whistle, music) and verbal commands. | not engaged in regular practice of physical activity | 60mins a session, 3 times a week for four months. | MMSE, | CDT,  BBS,  TUG,  MMSE | NA | physical activity with dual task seems to have contributed to the maintenance and improvement of the motor and cognitive functions |  |
| Rolland[12]  (2007)  France | EG:82.8(7.8) CG:83.1(7.0) | EG:67 CG:67 | aerobic, strength, flexibility, and balance training. | routine medical care | 1 hour a session, twice a week, total 88 sessions in 12 months. | NINDS-  ADRDA,  MMSE | MMSE, NPI,  ADL | NA | A simple exercise program, 1 hour twice a week, led to significantly slower decline in ADL score in patients with AD living in a nursing home than routine medical care |  |
| Sobol [13] (2016)  Denmark | EG:69.8(7.4) CG:71.3(7.3) | EG:107 CG:93 | moderate-to-high–intensity aerobic exercise on ergometer bicycle, cross trainer, and treadmill | usual treatment | 60mins a session, 3 times a week, for 4 months | NINDS-  ADRDA | SDMT, NPI, TUG, 10-m walk test | NCT01681602 | Aerobic exercise showed significant positive effects on physical performance. |  |
| Steinberg[14]  (2009)  USA | EG:76.5(3.9) CG:74.0(8.1) | EG:14 CG:13 | Aerobic fitness: brisk walking (moderate-intensity activity); Strength training; Balance  and flexibility training. | home safety  assessment. | at least 10 hours per week. 3 times a week for 12 weeks. | NINDS-  ADRDA,  MMSE | MMSE, NPI,  ADL | NA | exercise group demonstrated a trend for improved performance on measures of hand function and lower extremity strength. On secondary outcome measures, trends toward worse depression and lower quality of life ratings were noted. |  |
| Venturelli[15]  (2011)  Italy | EG:83(6) CG:85(5) | EG:12 CG:12 | maintain a constant walking speed and to avoid accelerating or stopping | daily organized activities | a minimum of 30 minutes of moderate exercise (walking) 4 times a week, lasting 6 months | CDR,  MMSE | MMSE,  ADL,  POMA,  PPT | NA | it is possible to stabilize the progressive cognitive dysfunctions through a specific walking program. |  |
| Vidoni[16]  (2019)  USA | EG:74.1(6.8) CG:71.1(8.8) | EG:33 CG:32 | a weekly goal of 60 minutes of aerobic exercise in week 1 and increased duration by approximately 21 minutes per week until to 150 minutes weekly, HR zones were gradually increased from 40–55% to 60–75% | Non-aerobic exercises (core strengthening, resistance bands, modified tai chi or yoga) | 150 minutes per week, distributed over 3 to 5 sessions, lasting 26 weeks | CDR | DAD,  ADL | NCT01129115 | Our analysis extends recent work by revealing specific benefits for instrumental activities of daily living for individuals in the early stages of AD and supports the value of exercise for individuals with cognitive impairment. |  |
| Vreugdenhil[17]  (2012)  Australia | EG:73.5  (51-83) CG:74.7  (58-89) | EG:20 CG:20 | body strength and balance training, brisk walking | routine medical care | a 4-month at-home exercise programme, at least 30 minutes of brisk walking everyday | NINDS-  ADRDA,  DSM-IV | ADAS-Cog,  MMSE,  TUG,  ADL | NA | A community-based exercise programme can improve cognitive and physical function and independence in ADL |  |
| Yágüez[18]  (2011)  UK | EG:70.5(8.0) CG:75.7(6.9) | EG:15 CG:12 | Brain Gym movements are designed to activate balanced and equal muscles on both sides of the body. Stretching different parts of the body, circular movements of the extremities and isometric tensions of muscles groups. | provided psychological support, creating a friendly and supportive environment and encouraging group interaction. | with weekly sessions of 2 h, for 6 weeks | ICD-10,  MMSE | CANTAB-Expedio, | NA | a short course of non-aerobic movement based exercise is already effective at least in some aspects of cognitive functioning in patients with AD. |  |
| Yang[19]  (2015)  China | EG:72.0(6.7) CG:71.9(7.3) | EG:25 CG:25 | cycling training at 70% of MHR | No intervention,  health education | maximal intensity for 40 min/d, 3 d/wk for 3 months. | MMSE,  NINDS-  AIREN | MMSE, NPI, Qol-AD,  ADAS-Cog | NA | moderate intensity of  aerobic exercise can improve cognitive function in patients with mild Alzheimer’s disease. |  |
| Yu [20] (2021)  USA | EG:77.4(6.6) CG:77.5(7.1) | EG:64 CG:32 | cycling at 50–75% of heart rate reserve (HRR) | stretching and range-of-motion,<20% of HRR | 20-50mins a session, 3 times a week, for 6 months | CDR,  MMSE | ADAS-cog, composite scores | NCT01954550 | The 6 month change in ADAS-cog sinificnatly less than the natural increase in AD |  |
| Chunhong [21] (2015)  China | EG:70.7(7.4) CG:70.2(8.5) | EG:27 CG:30 | Aerobic exercise, 50%-70% of HRR | usual treatment | 60-90mins, 3 times a week, for 4 month | DSM-IV, MMSE | MMSE Qol-AD ASCS-ADL | NA | Aerobic training therapy can significantly improve the cognition, ADL and quality of life. |  |
| Qing [22] (2020)  China | EG:74(11) CG:70(11) | EG:30 CG:30 | Aerobic exercise, 60%-80% of HRR | health education | 40mins a session, 3 times a week, for 3 months | NINDS-  ADRDA | MMSE, NPI, Qol-AD, ASCS-ADL, BBS | NA | MMSE, ADCS-ADL scores are significantly higher in aerobic exercise group at 3 months follow-up |  |
| Yin[23]  (2017)  China | EG:70.9(9.2) CG:70.3(7.7) | EG:24 CG:24 | Stepping breathing, head movement, lateral movement, body transport, training at 70% of MHR | daily organized activities | 40mins a session, 3 times a week, for 3 months | DSM-IV, MMSE | MMSE, Qol-AD,  ADAS-Cog | NA | Aerobic exercise can improve cognitive function, especially memory function, mental and behavioral symptoms of AD patients. |  |
| Haiyan[24]  (2016)  China | EG:72.9(5.4) CG:73.7(4.6) | EG:39 CG:39 | 400 meter's brisk walking, | daily organized activities, health education | At least 60mins daily, 3 times a week, for 4 months | CDR,  NINDS-ADRDA | MMSE, NPI | NA | Aerobic exercise can improve the daily living ability and memory ability of patients with mild to moderate AD, and improve the mental symptoms of patients. |  |
| Shiyan[25]  (2014)  China | EG:70.3(7.2) CG:71.1(8.2) | EG:21 CG:20 | Training with cycle ergometer exercise at 70% of MHR | daily organized activities | 40mins a session, 3 times a week, for 3 months | MMSE,  MRI/CT,  NINDS-  AIREN | MMSE,  ADAS-Cog | NA | Moderate-intensity aerobic exercise can improve cognitive and motor function in AD patients. |  |
| Wei[26]  (2014)  China | EG:71.2(7.0) CG:70.0(8.9) | EG:26 CG:28 | cycling at 70% of MHR | daily organized activities | 40mins a session, 3 times a week, for 3 months | DSM-IV,  NINDS-ADRDA,  MMSE | MMSE, ADAS-Cog Qol-AD, ASCS-ADL, | NA | Medication-assisted moderate-intensity aerobic training program used in this study can improve cognitive function and activities of daily living in AD patients. |  |
| Ying[27]  (2014)  China | EG:71.6(5.8)  EG:70.8(8.5) CG:70.6(8.4) | EG:7  EG:7 CG:19 | cycling at 50%-70% of MHR | daily organized activities | 40mins a session, 3 times a week, for 3 months | NINDS-ADRDA,  MMSE | MMSE, ADAS-Cog, ASCS-ADL | NA | Aerobic exercise therapy can significantly improve the cognitive function and neurologic symptoms of AD patients, but has no effect on the ability of daily living activities. |  |
| Lanyun[28]  (2015)  China | EG:72.1(6.1)  EG:71.5(5.9) CG:70.6(7.3) | EG:18  EG:18 CG:18 | Training with cycle ergometer exercise at 70% of MHR | usual treatment | 30mins - 40mins a session, 3-4 times a week, for 6 months | DSM-IV,  NINDS-ADRDA,  MMSE | MMSE, NPI,  ADAS-Cog | NA | Aerobic exercise is more beneficial in improving cognitive function, living ability and neuropsychiatric symptoms in AD patients. |  |
| EG: experiment group; CG: contral group; SD: standerd deviation; ADAS-cog: Alzheimer's Disease Assessment Scale - Cognition; ADCS-ADL: Alzheimer's Disease Cooperative Study - Activity of Daily Living; BBS: Berg Balance Scale; CDR:Clinical Dementia Rating; CSD: Cornell Scale for Depression in Dementia; DSM-IV: Diagnostic and Statistical Manual of Mental Disorders, fourth edition; DAD: Disability Assessment for Dementia; ERFC: Rapid Evaluation of Cognitive Function; FACS: Functional Assessment of Communication Skills for Adults; LPRS: London Psychogeriatric Rating Scale; SDMT: Symbol Digit Modalities Test; CANTAB-Expedio: The Cambridge Neuropsychological Test Automated Battery; NPI: Neuropsychiatric Inventory; MMSE: Minimum Mental State Examination; POMA: Performance Oriented Mobility Assessment; PPT: physical performance test; Qol-AD: Quality of Life - Alzheimer's Disease; NINDS-ADRDA: National Institute of Neurological and Communicative Disorders and Stroke and the Alzheimer's Disease and Related Disorders Association; NA: Not available; TUG: Time Up and Go test; VF: Verbal Fluency test. @bias risk evaluation. Symbol means: unclear, high risk, low risk. The seven evaluation items included random cohort allocation, allocation hiding, double blinding of participants and staff, blinded evaluation results, complete report data, selective reporting and others. * record at baseline | | | | | | | | | | |

**References:**

1. Aguiar P, Monteiro L, Feres A, Gomes I, Melo A. Rivastigmine transdermal patch and physical exercises for Alzheimer's disease: a randomized clinical trial. Curr Alzheimer Res 11 (6): 532-7, 2014.

2. Cott CA, Dawson P, Sidani S, Wells D. The effects of a walking/talking program on communication, ambulation, and functional status in residents with Alzheimer disease. Alzheimer Dis Assoc Disord 16 (2): 81-7, 2002.

3. de Oliveira SF, Ferreira JV, Plácido J, Sant'Anna P, Araújo J, Marinho V, et.al. Three months of multimodal training contributes to mobility and executive function in elderly individuals with mild cognitive impairment, but not in those with Alzheimer's disease: A randomized controlled trial. Maturitas 126: 28-33, 2019.

4. Fajersztajn L, Cordeiro RC, Andreoni S, Garcia JT. Effects of functional physical activity on the maintenance of motor function in Alzheimer's disease. Dementia & Neuropsychologia 2 (3): 233-240, 2008.

5. Hoffmann K, Sobol NA, Frederiksen KS, Beyer N, Vogel A, Vestergaard K, et.al. Moderate-to-High Intensity Physical Exercise in Patients with Alzheimer's Disease: A Randomized Controlled Trial. J Alzheimers Dis 50 (2): 443-53, 2016.

6. Holthoff VA, Marschner K, Scharf M, Steding J, Meyer S, Koch R, et.al. Effects of Physical Activity Training in Patients with Alzheimer’s Dementia: Results of a Pilot RCT Study. PLOS ONE 10 (4): e0121478, 2015.

7. Kemoun G, Thibaud M, Roumagne N, Carette P, Albinet C, Toussaint L, et.al. Effects of a physical training programme on cognitive function and walking efficiency in elderly persons with dementia. Dement Geriatr Cogn Disord 29 (2): 109-14, 2010.

8. Lamb SE, Sheehan B, Atherton N, Nichols V, Collins H, Mistry D, et.al. Dementia And Physical Activity (DAPA) trial of moderate to high intensity exercise training for people with dementia: randomised controlled trial. BMJ 361: k1675, 2018.

9. Morris JK, Vidoni ED, Johnson DK, Van Sciver A, Mahnken JD, Honea RA, et.al. Aerobic exercise for Alzheimer's disease: A randomized controlled pilot trial. PLoS One 12 (2): e0170547, 2017.

10. Öhman H, Savikko N, Strandberg TE, Kautiainen H, Raivio MM, Laakkonen M, et.al. Effects of Exercise on Cognition: The Finnish Alzheimer Disease Exercise Trial: A Randomized, Controlled Trial. Journal of the American Geriatrics Society 64 (4): 731-738, 2016.

11. Pedroso RV, Coelho FG, Santos-Galduróz RF, Costa JL, Gobbi S, Stella F. Balance, executive functions and falls in elderly with Alzheimer's disease (AD): a longitudinal study. Arch Gerontol Geriatr 54 (2): 348-51, 2012.

12. Rolland Y, Pillard F, Klapouszczak A, Reynish E, Thomas D, Andrieu S, et.al. Exercise program for nursing home residents with Alzheimer's disease: a 1-year randomized, controlled trial. J Am Geriatr Soc 55 (2): 158-65, 2007.

13. Sobol NA, Hoffmann K, Frederiksen KS, Vogel A, Vestergaard K, Brændgaard H, et.al. Effect of aerobic exercise on physical performance in patients with Alzheimer's disease. Alzheimers Dement 12 (12): 1207-1215, 2016.

14. Steinberg M, Leoutsakos JM, Podewils LJ, Lyketsos CG. Evaluation of a home-based exercise program in the treatment of Alzheimer's disease: the Maximizing Independence in Dementia (MIND) study. Int J Geriatr Psychiatry 24 (7): 680-5, 2009.

15. Venturelli M, Scarsini R, Schena F. Six-month walking program changes cognitive and ADL performance in patients with Alzheimer. Am J Alzheimers Dis Other Demen 26 (5): 381-8, 2011.

16. Vidoni ED, Perales J, Alshehri M, Giles AM, Siengsukon CF, Burns JM. Aerobic Exercise Sustains Performance of Instrumental Activities of Daily Living in Early-Stage Alzheimer Disease. J Geriatr Phys Ther 42 (3): E129-E134, 2019.

17. Vreugdenhil A, Cannell J, Davies A, Razay G. A community-based exercise programme to improve functional ability in people with Alzheimer’s disease: a randomized controlled trial. Scandinavian Journal of Caring Sciences 26 (1): 12-19, 2012.

18. Yágüez L, Shaw KN, Morris R, Matthews D. The effects on cognitive functions of a movement-based intervention in patients with Alzheimer's type dementia: a pilot study. Int J Geriatr Psychiatry 26 (2): 173-81, 2011.

19. Yang SY, Shan CL, Qing H, Wang W, Zhu Y, Yin MM, et.al. The Effects of Aerobic Exercise on Cognitive Function of Alzheimer's Disease Patients. CNS Neurol Disord Drug Targets 14 (10): 1292-7, 2015.

20. Yu F, Vock DM, Zhang L, Salisbury D, Nelson NW, Chow LS, et.al. Cognitive Effects of Aerobic Exercise in Alzheimer’s Disease: A Pilot Randomized Controlled Trial. Journal of Alzheimer's Disease 80 (1): 233-244, 2021.

21. Chunhong Chang, Wei Wang, Yi Zhu, Siyu Yang, Haiyan Li, Qin Wang, et.al. The effect of aerobic training on Alzheimer's disease. Chinese Journal of Rehabilitation Medicine 30 (11): 1131-1134+1161, 2015.

22. Qing He, Yi Xie, Tong Wang, Li Shao, Jingcui Qin, Yan Lu, et.al. Analysis of the Effect and Individual Differences of Aerobic Exercise on Alzheimer's Disease. Chinese Trauma and Disability Medicine 28 (24): 16-20, 2020.

23. Yin Liu, Tong Wang, Yi Zhu, Wei Wang, Mengmei Yin, Wanrong Zhang, et.al. A study of moderate-intensity aerobic exercise for improving the cognitive function of patients with Alzheimer's disease. China Rehabilitation 32 (5): 386-389, 2017.

24. Haiyan Mu, Jihui Lu, Zhihui Hao, Wenjie Li, Mo Li. The effect of aerobic exercise on the living ability, cognitive function and mental symptoms of patients with mild to moderate Alzheimer's disease. Chinese Journal of Multiple Organ Diseases in the Elderly 15 (6): 451-454, 2016.

25. Shiyan Wang, Yi Zhu, Qin Zhang, Yabei Fan, Jianguo Sun, Ting Wu, et.al. Effects of moderate-intensity aerobic exercise on the cognitive and motor function of patients with Alzheimer's disease. Chinese Journal of Physical Medicine and Rehabilitation 36 (10): 765-768, 2014.

26. Wei Wang, Yi Zhu, Siyu Yang, Chunhong Chang, Ying Wang, Haiyan Li, et.al. Effects of aerobic training on cognitive function and activities of daily living in patients with Alzheimer's disease. China Journal of Rehabilitation Medicine 29 (12): 1151-1155, 2014.

27. Ying Wang, Feifei Shen, Yi Zhu, Siyu Yang, Haiyan Li, Qin Wang, et.al. A clinical study of moderate to high intensity aerobic exercise intervention in Alzheimer's disease. Chinese Clinical Neuroscience 0 (5): 504-509, 2014.

28. Lanyun Yan, Wei Wang, Feifei Shen, Siyu Yang, Wei Wang, Qin Wang, et.al. A clinical study on the intervention of aerobic exercise with different training time in mild to moderate Alzheimer's disease. Chinese Journal of Rehabilitation Medicine 30 (8): 771-776, 2015.
